# Real-Time Sensor Networks and Systems for the Industrial IoT: What Next?

**DOI:** 10.3390/s20185023

**Published:** 2020-09-04

**Authors:** Christos Koulamas, Mihai T. Lazarescu

**Affiliations:** 1 Industrial Systems Institute, Athena Research Center, PSP Bldg, Stadiou Strt, 26504 Patras, Greece; 2 Dipartimento di Elettronica e Telecomunicazioni, Politecnico di Torino, I-10129 Turin, Italy; mihai.lazarescu@polito.it

## 1. Introduction

The Industrial Internet of Things (Industrial IoT—IIoT) is the emerging core backbone construct for the various cyber-physical systems constituting one of the principal dimensions of the 4th Industrial Revolution. While initially born as a concept inside specific industrial applications of the generic IoT technologies, for the optimization of the operational efficiency in automation and control, it quickly became the vehicle for the achievement of the total convergence of Operational Technologies (OT) with Information Technologies (IT). Today, it is already breaking the traditional borders of automation and control functions in the process and manufacturing industry, towards a wider domain of functions and industries, embraced under the dominant, global initiatives and architectural frameworks of Industry 4.0 (or Industrie 4.0) in Germany, Industrial Internet in the US, Society 5.0 in Japan, and Made-in-China 2025 in China. As real-time embedded systems are quickly achieving ubiquity in people’s everyday life and in industrial environments, and many processes already depend on real-time cyber-physical systems and embedded sensors, IoT integration with cognitive computing and real-time data exchanges is essential for real-time analytics and realization of digital twins in smart environments and services under the various frameworks’ provisions [1,2]. In this context, real-time sensor networks and systems for the Industrial IoT encompass multiple technologies and raise significant design, optimization, integration, and exploitation challenges.

## 2. The Current Issue

The ten articles in this Special Issue provide advances of real-time sensor networks and systems that are significant enablers of the Industrial IoT paradigm. In the relevant landscape, the wireless networking technologies domain is centrally positioned, as expected. Seferagić et al. comparatively discuss various alternatives for the engineering and deployment of industrial wireless sensor and actuator networks (IWSAN) in [3]. In this work, LoRa, IEEE 802.11ah (Wi-Fi HaLow), Narrowband-IoT (NB-IoT), WirelessHART, ISA100.11a, Bluetooth Low Energy (BLE), and IEEE 802.15.4g with IEEE 802.15.4e TSCH are reviewed and compared over multiple axes, including communication range, latency, reliability, data rates, energy consumption, scalability, and spectrum regulations. WSAN reliability and real-time performance are key aspects for effective and stable wireless control, and nearly half of the articles of this Special Issue focus on the optimization of the quality of service offered by the wireless stack. In this view, Park et al. propose and evaluate in [4] three different wireless transmission scheduling schemes serving multiple and heterogeneous control systems. The reported results suggest that the centralized Lyapunov-based scheduling approach stands closer to the ideal solution, and that the distributed random access is a good candidate for control systems with a small number of control loops. Furthermore, multipath retransmission in multiple channel deterministic wireless networks, such as WirelessHART, are discussed by Wang et al. in [5], proposing the CEM-RM resource-scheduling algorithm. Through simulations as well as data collected from real experimental setups, the authors demonstrate improved performance in terms of schedulability, end-to-end delay, and resource usage efficiency compared to the M-LLF and M-RM scheduling policies. The WirelessHART protocol also provides the context for Wu et al.’s research in [6], and especially the priority assignment for real-time data flows mapped over the TDMA operation at the MAC layer. Cheng et al. address soft real-time industrial applications over IEEE 802.11ah in [7]. In this work, the authors propose the channel-aware contention window adaption (CA-CWA) algorithm, which improves the packet loss rate and average delay by adapting the contention window based on the channel status. Along the wireless network technologies, legacy wired real-time networks can play significant roles in the modern IIoT landscape, as shown by Lee et al. in [8]. The authors propose a lightweight CAN virtualization technology for virtual controllers containerized on top of an OS-based virtualization technology that provides virtual CAN interfaces and buses at the device driver level.

Several articles in this Special Issue address important industrial IoT security and infrastructure protection issues. Tedeschi et al. analyze the integration of modern IoT and legacy industrial equipment for real-time machine condition monitoring applications, and the resulting security challenges in [9]. The authors propose a security-by-design approach that introduces real-time adaptation features for device security through subsystem isolation and lightweight authentication. Similarly, the protection of legacy devices in critical infrastructures is addressed by Fournaris et al. in [10]. The proposed dedicated hardware security token (HST) supports a secure event log and real-time monitoring mechanism for anomaly detection, thus isolating the typically insecure legacy OS logging mechanisms. In connection to this, Wielgosz et al. introduce in [11] AI concepts in real-time smart sensors to detect anomalies for the protection of superconducting industrial machinery. In particular, an embedded Recurrent Neural Network (RNN) has been designed for a device protecting the main subsystem of CERN’s Large Hadron Collider (LHC) accelerator. The proposed scheme demonstrates a low memory footprint and architectural uniformity, allowing an efficient hardware implementation and a distributed edge-computing cluster of sensors that reduce resource consumption, latency, and throughput. AI is also the core technological domain exploited by Ntalianis et al. in [12], which is an excellent example of the extended scope of today’s Industrial IoT. Specifically, the authors propose a deep CNN sparse coding used for the classification of inhaler sounds in real-time directly on the time-domain, thus avoiding computationally expensive feature extraction techniques and allowing for an efficient implementation on constrained hardware and the integration with real-time IIoT technologies.

## 3. What Next?

The real-time performance of the evolving IIoT networks and systems is increasingly considered among the major IIoT challenges, together with energy efficiency, security, and interoperability [13]. Coming as a challenging requirement from various application domains, real-time operation is also recognized as a mitigator of other horizontal challenges, such as energy efficiency or reliability. For example, the TSCH operation of IEEE 802.15.4e is quite often proposed either as an energy efficiency measure, since nodes can sleep until their precise communication slot time, or as a reliability enhancement, through its inherent frequency hopping capability, which still requires perfect time synchronization [14]. Furthermore, timeliness and security emerge as two major driving forces behind the increasing shift of processing towards the edge, tightly coupled with AI capabilities in constrained embedded devices. The components needed to build real-time wireless sensor network segments are already widely available. However, there is still open space for tools and mechanisms that will master the complexity of configuring the scheduling functions and other relevant specificities, including secure bootstrapping of extremely constrained structures in an automatic or semi-automatic way, enabling both specialists and non-specialists to deploy and exploit these new technologies. Most importantly though, the challenge consists in providing end-to-end timeliness guarantees in complex paths of heterogeneous sensor networks and systems, that may also include heavier real-time data analytics services over virtualized cloud infrastructures.

For all these reasons, we expect that the pressure for solutions will be increased with the upcoming penetration of 5G [15] and the deployment of more and more complex infrastructures for critical monitoring and control, either inside the traditional process and manufacturing industry, or outside it, in modern IIoT verticals, such as autonomous vehicles and V2V/V2I interactions, smart agriculture, smart energy, smart buildings and cities, and many others.
